# Supplementary material for: Physical origin of higher-order soliton fission in nanophotonic semiconductor waveguides
Source: Sci Rep. 2018 Nov 21;8:17177. doi: 10.1038/s41598-018-34344-4 (PMC6249288; doi:10.1038/s41598-018-34344-4)
Supplement: Supplementary file 1 — Supplementary Information [file 41598_2018_34344_MOESM1_ESM.pdf]

Supplementary Information – Physical origin of higher-order soliton fission in nanophotonic semiconductor waveguides

Charles Ciret,[1,2] Simon-Pierre Gorza,[3] Chad Husko,[4] Gunther Roelkens,[5] Bart Kuyken,[5] and François Leo[3,*]

[1]OPERA-Photonics, Université libre de Bruxelles (ULB), 50 Av. F. D. Roosevelt, CP 194/5, B-1050 Brussels, Belgium

[2] Laboratoire de Photonique dAngers EA 4464, Universite dAngers, 2 Boulevard Lavoisier, 49000 Angers, France

[3]OPERA-Photonics, Universit libre de Bruxelles (ULB), 50 Av. F. D. Roosevelt, CP 194/5, B-1050 Brussels, Belgium

[4]Center for Nanoscale Materials, Argonne National Laboratory, Argonne, IL 60439

[5]Photonics Research Group, Department of Information Technology, Ghent University-IMEC, B-9000 Ghent, Belgium

This article contains two supplementary figures for the manuscript entitled "Physical origin of higher-order soliton fission in nanophotonic semiconductor waveguides".

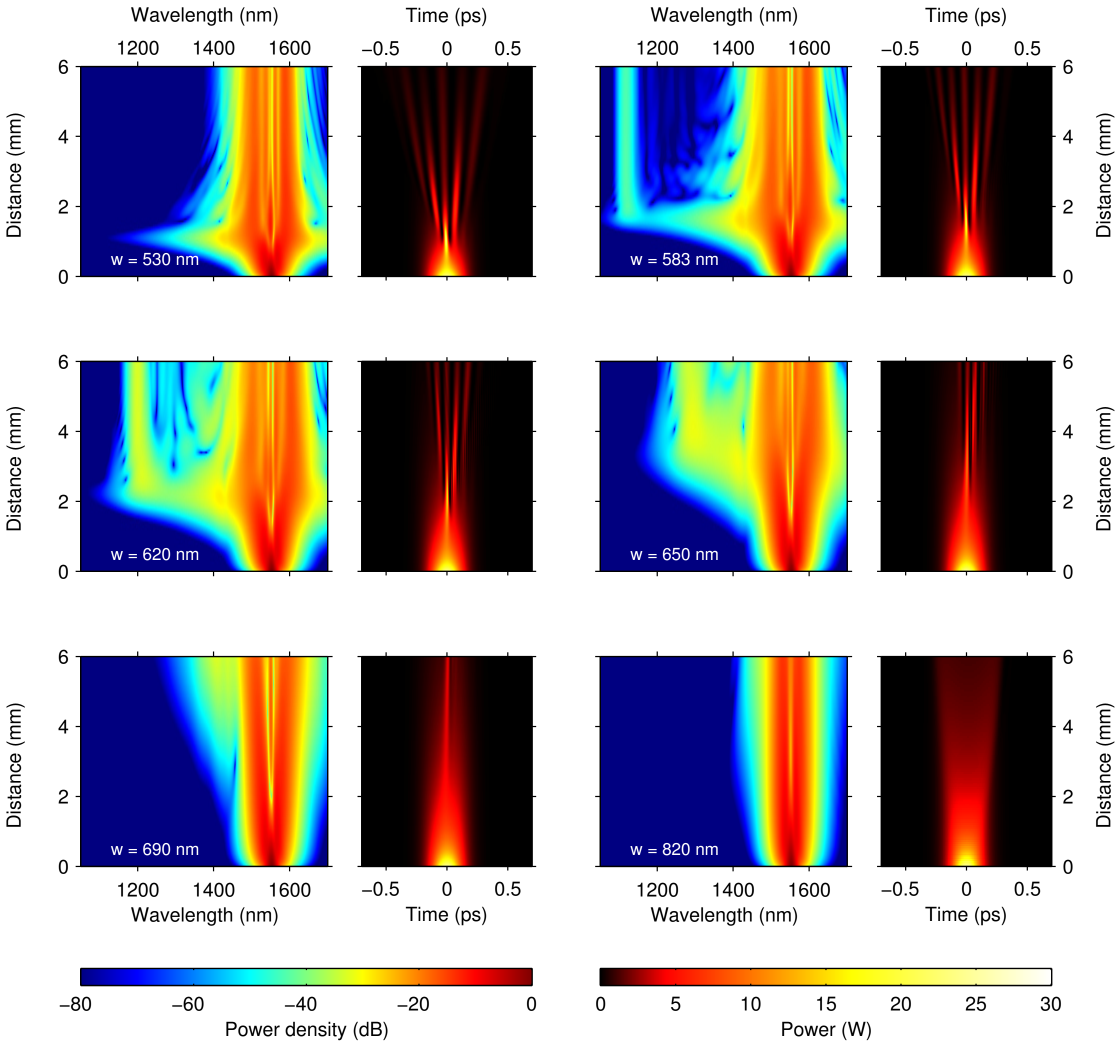

FIG. S1: Pseudocolor plots of the simulated spetra (left) and temporal (right) evolution along the different waveguides for a 165 fs (FWHM) 30 W sech input pulse. The width has been chosen so as to maximiz the agreement with our expeirmental results. See Figure 1 of the manuscript.

*Electronic address: francois.leo@ulb.ac.be

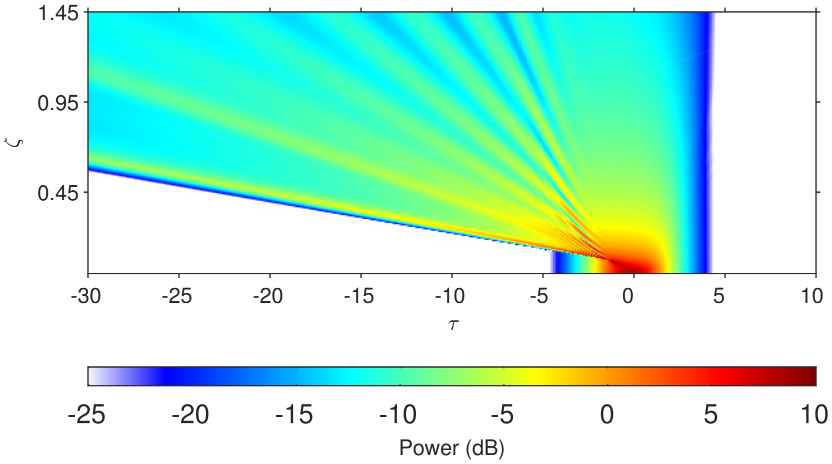

FIG. S2: Colormap of the propagation through a silicon waveguide, as simulated with the normalized model [Equation (2)] for a $N = 6$ input soliton. ($\eta = 100, \alpha_{2PA} = 58\ \mathrm{W^{-1}m^{-1}}$, $\gamma = 323\ \mathrm{W^{-1}m^{-1}}$).
